# Valve-sparing procedure for acute aortic regurgitation due to intimal intussusception in a minimally localized aortic root dissection

**DOI:** 10.1186/s44215-024-00180-4

**Published:** 2024-12-20

**Authors:** Takuya Matsushiro, Tomoki Tamura, Daiki Ishiwaki, Takumi Umibe, Nobuyuki Inoue

**Affiliations:** Department of Cardiovascular Surgery, National Center for Global Health and Medicine, Toyama 1-21-1, Shinjuku, Tokyo, Japan

**Keywords:** Intimo-intimal intussusception, Localized aortic root dissection, Valve sparing

## Abstract

**Background:**

Acute heart failure due to aortic regurgitation (AR) is a severe comorbidity of type A acute aortic dissection (AAD). Valve-sparing aortic root replacement is typically performed when the aortic valve remains intact.

**Case presentation:**

A 33-year-old male presented to our hospital with chest pain. Initial computed tomography (CT) scans did not clearly identify an aortic dissection. However, subsequent evaluations suggested acute coronary syndrome. Catheter angiography revealed difficulties in catheterizing the coronary arteries, and echocardiography detected aortic insufficiency. Electrocardiogram-gated CT angiography ultimately confirmed a localized aortic root dissection, necessitating urgent surgical intervention. The patient underwent valve reimplantation to preserve the aortic valve. The postoperative course was uneventful, with follow-up echocardiography and CT showing no residual dissection or regurgitation.

**Conclusion:**

This report highlights a case of acute aortic root dissection resulting in acute AR. The primary cause of AR in this case was the intussusception of the disrupted aortic intima. The dissection was confined solely to the aortic root. The patient underwent successful valve reimplantation, with no postoperative complications. Electrocardiogram-gated CT angiography and transesophageal echocardiography proved valuable in identifying localized aortic abnormalities with precision.

## Background

Acute type A aortic dissection (ATAAD) is a life-threatening condition that requires prompt surgical intervention. Among its most severe comorbidities is acute heart failure caused by aortic regurgitation (AR). The primary surgical treatment for ATAAD involving the aortic root is root replacement with a prosthetic valve, commonly known as the Bentall procedure. While mechanical valves are often used in younger patients, they necessitate lifelong anticoagulant therapy. As a result, the valve-sparing aortic root replacement (VSARR) procedure is a preferable option for younger patients with ATAAD. This report details a case of acute AR caused by aortic root dissection, which was successfully managed with VSARR.

## Case presentation

A 33-year-old male presented to the emergency department of another hospital with chest pain. He had a history of minimal change disease during his teenage years, successfully treated at that time. Since then, annual health checkups had revealed no abnormalities, including hypertension. Additionally, the patient did not exhibit any physical features typically associated with Marfan syndrome. Initial computed tomography (CT) scans failed to provide clear evidence of aortic dissection. He was subsequently referred to our hospital.

**Fig. 1:**
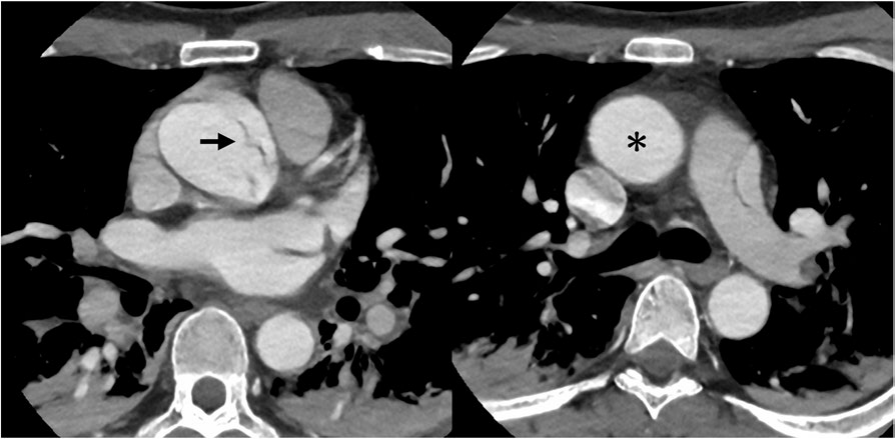
Preoperative ECG-gated CT angiography shows that the intimal flap (black arrow) is in the aortic root (**a**), while the ascending aorta (astarisk) is intact (**b**)

**Fig. 2:**
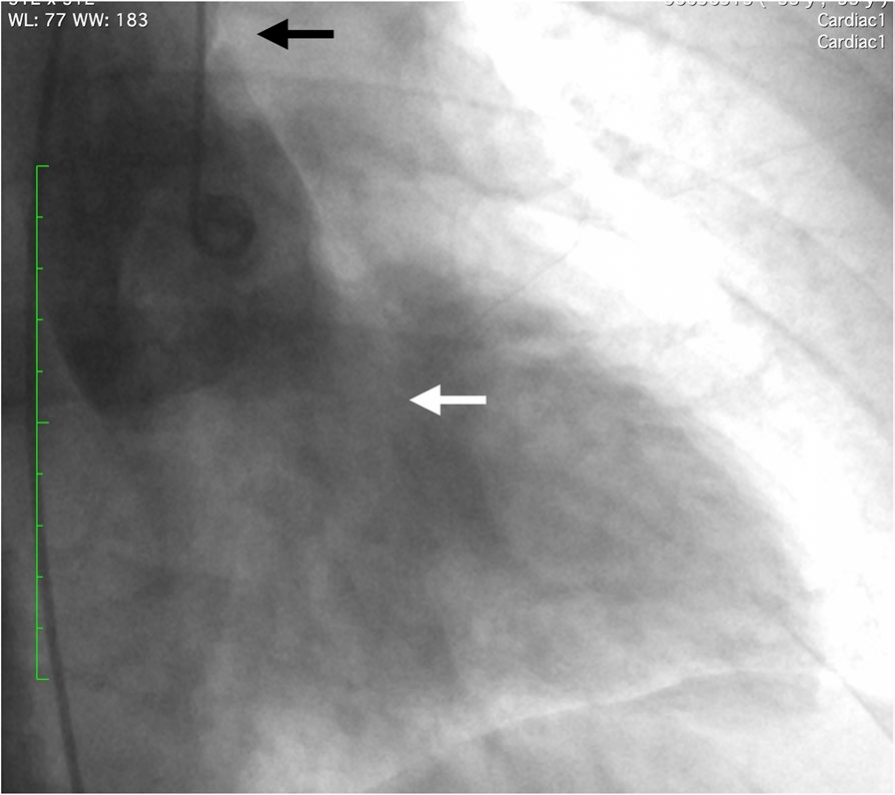
The angiography of the aorta shows the disrupted aortic intima (black arrow) and severe aortic regurgitation

On admission, electrocardiography (ECG) showed ST depression in leads II, III, and aVF, while blood tests indicated a slightly elevated creatinine kinase level. These findings initially suggested acute coronary syndrome. Bedside echocardiography revealed severe aortic insufficiency, though no intimal flap was detected in the ascending aorta or the sinus of Valsalva. Subsequently, ECG-gated CT angiography identified a disruption in the continuity of the aortic intima, although no false lumen was apparent (Fig. 1). While these CT findings pointed to type A aortic dissection, the possibility of acute coronary syndrome was still considered based on the ECG and blood test results. Catheter angiography was performed to exclude coronary artery thromboembolism (Fig. 2), but both ostial coronary arteries could not be catheterized during the procedure. After a comprehensive evaluation, the patient was diagnosed with acute aortic dissection at the aortic root leading to acute AR.

**Fig. 3:**
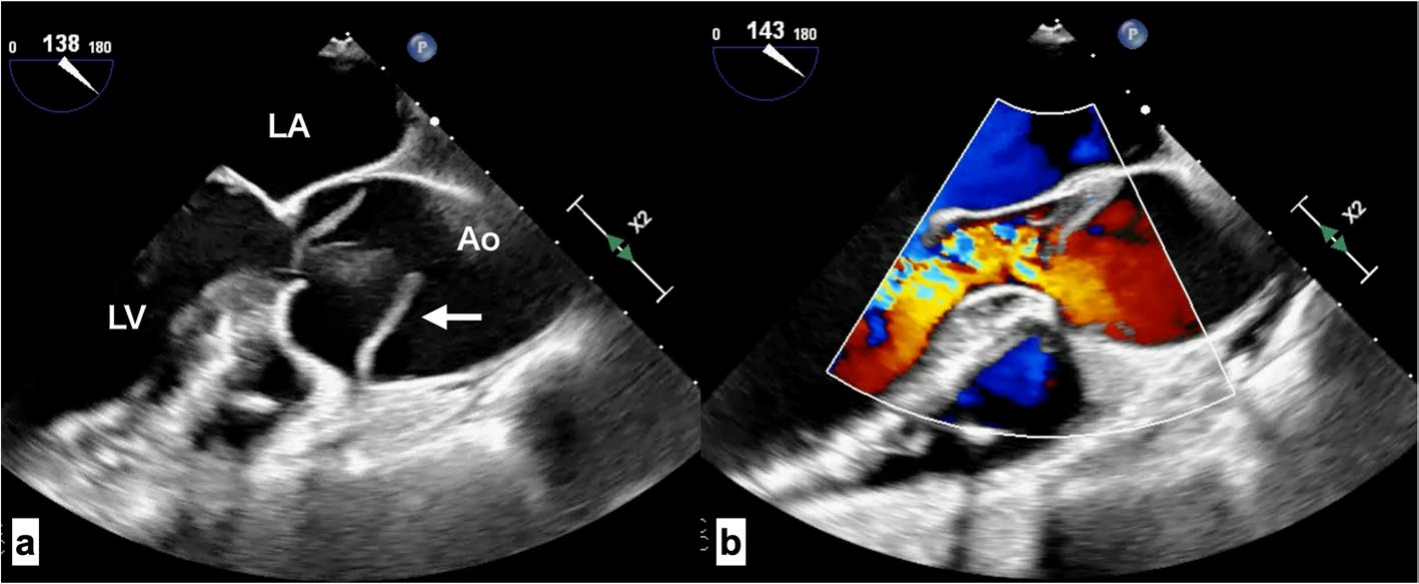
The transesophageal echocardiogram during the surgery shows the intimal flap (**a**) (white arrow), which is inverted into left ventricle which causes severe AR (**b**)

**Fig. 4:**
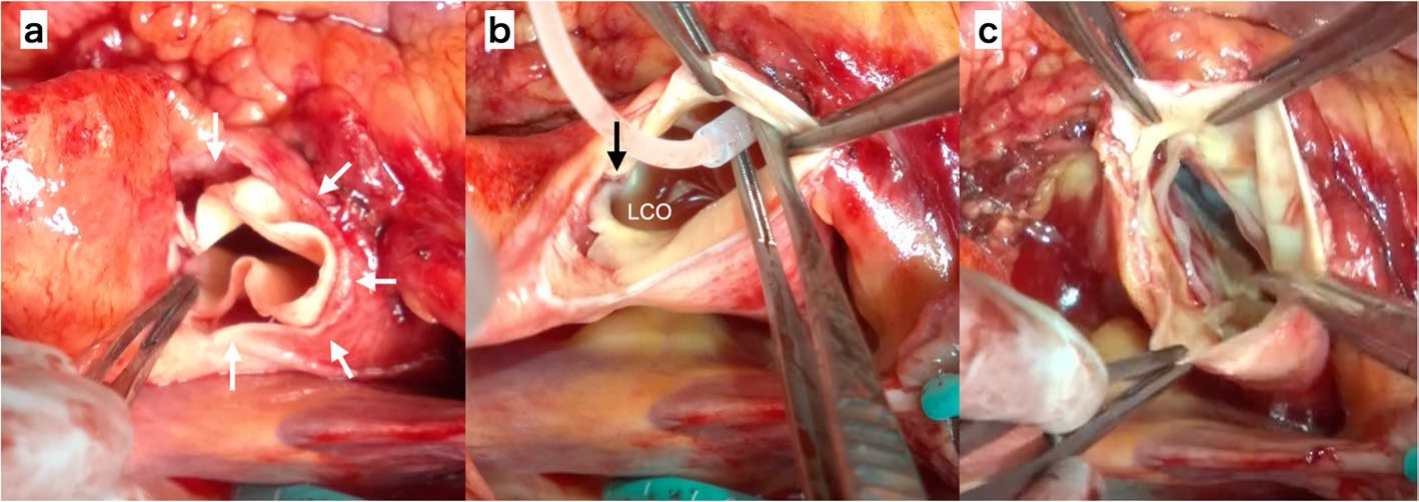
Intraoperative photographs present the dissection which is extended circumferentially (white arrow) (**a**) and intimal tear (black arrow) which extends immediately adjacent to the left coronary ostium (**b**). The aortic valve is intact, and the coaptation of three leaflets is good (**c**)

The patient was transferred to the operating room for emergency surgery. Cardiopulmonary bypass was established via right atrial drainage with return through the right femoral artery. Intraoperative transesophageal echocardiography revealed that the dissection was localized to the aortic root, with the distal ascending aorta, 2–3 cm above the sinotubular junction, remaining intact (Fig. 3). The diameter of the sinus of Valsalva was slightly enlarged at 45 mm. The ascending aorta was cross-clamped, and cardioplegia was administered via an aortic root cannula. Upon transecting the ascending aorta, a torn intima was found immediately adjacent to the left coronary ostium (Fig. 4). The dissection encircled the entire aortic circumference, and the disrupted intima had inverted toward the left ventricle, causing aortic valve insufficiency. The aortic valve itself was intact, with good coaptation of the three cusps. To retain his own aortic valve, a reimplantation procedure known as the “David procedure” was performed. Graft sizing was based on measurements of the noncoronary (NC) and left coronary (LC) commissure heights. The aortic valve and annulus were excised from the aortic wall, leaving a 5-mm sewing margin. The preserved valve was securely sutured into a 26-mm Gelweave Valsalva graft (VASCUTEK Ltd., Scotland, UK), with the intima and adventitia fixed within the 5-mm sewing margin. No glue was used for fixation. The three commissures were attached to the junction between the skirt (with vertical crimping) and the body (with horizontal crimping) of the graft. The intimal tear extended circumferentially near the left coronary artery orifice, necessitating the excision of the left coronary button along the tear line, which was subsequently anastomosed to the graft without issue.

Postoperative recovery was uneventful, with no significant complications. An echocardiogram performed 1 week after surgery showed no residual AR, and contrast-enhanced CT confirmed the absence of residual aortic dissection. Pathological examination of the resected aortic wall revealed no abnormalities.

## Discussion and conclusions

The risk of aortic root reconstruction surgery for acute type A aortic dissection (ATAAD) remains significant. In Japan, the 30-day mortality rate for aortic root surgery in ATAAD patients was 11.7% in 2021, notably higher than those for aortic arch surgery (6.8%) and ascending aorta surgery (6.7%) [1]. Valve-sparing aortic root replacement (VSARR) involves two primary techniques: the “Remodeling” method introduced by Yacoub in 1983 [2] and the “Reimplantation” method developed by David in 1992 [3]. Anticoagulant therapy is unnecessary with VSARR as the native aortic valve leaflets are preserved. Current guidelines recommend these procedures for younger patients undergoing aortic root surgery [4].

In the present case, aortic regurgitation was attributed to intussusception of the circumferentially disrupted aortic intima. During diastole, the intimal flap prolapsed into the left ventricle, causing valve insufficiency. Notably, no structural abnormalities were observed in the valve leaflets. Aortic wall intussusception can also obstruct the coronary artery ostium, resulting in ECG changes. However, there was no evidence of coronary artery embolism, and coronary artery bypass was unnecessary. The intimal tear extended close to the left coronary ostium, necessitating the excision of the sinus of Valsalva. The intact leaflets and commissures of the aortic valve allowed effective readjustment and refixation, which significantly improved aortic insufficiency. In this case, VSARR was feasible. The surgery was performed using a previously described technique, with graft size determined by measuring the height of the noncoronary (NC) and left coronary (LC) commissures, as recommended by El Khoury and colleagues [5].

A PubMed search revealed a limited number of cases describing intussusception of the intimal flap, also known as “intimo-intimal intussusception,” due to ATAAD [6]. Only five case reports have detailed aortic dissections localized to the aortic root (Table 1) [7–11]. Similar to our case, localized aortic root dissection with intimal flap intussusception was challenging to diagnose at presentation. In some cases, a definitive diagnosis was delayed by more than 3 weeks after the initial presentation. This delay is partly due to the absence of a false lumen distal to the ascending aorta on contrast-enhanced CT. Dissection localized solely to the aortic root can be difficult to detect with contrast-enhanced CT because of artifacts caused by heart motion. ECG-gated CT can mitigate this issue and facilitate a definitive diagnosis. Additionally, transesophageal echocardiography is an effective modality for diagnosing minimally localized aortic root dissection. It should be considered when prior CT angiography fails to clearly identify the intimal flap or false lumen due to artifacts. However, the use of preoperative transesophageal echocardiography must be carefully evaluated, as it is an invasive procedure.

**Table 1:** Previous case reports of circumferential aortic dissection minimally located to aortic root

		**Age**	**Sex**	**Prior diagnosis**	**Definite diagnosis confirmed**	**Diagnose by**	**Surgery**
Yamabi H. et al. [7]	2011	62	Female	AR, normal aorta	NA	TEE	AAR + valve resuspention
Kanki H. et al. [8]	2013	34	Male	AR, normal aorta	On admission	TEE	Aortic valve repair
Ikeda A. et al. [9]	2017	46	Female	AR, normal aorta	NA	TEE	Bentall
Ito Y. et al. [10]	2018	43	Male	ACS with ST elevation	21 days later	TEE	Valve sparing
Yuge N. et al. [11]	2023	56	Male	ACS with ST elevation	34 days later	Coronary angiography	Bentall

## Data Availability

All relevant data are within the manuscript.

## Abbreviations

AR: Aortic regurgitation
ATAAD: Acute type A aortic dissection
CT: Computed tomography
ECG: Electrocardiogram
VSARR: Valve-sparing aortic root replacement
